# Efficacy and Safety of Chinese Medicinal Herbs for the Treatment of Hyperuricemia: A Systematic Review and Meta-Analysis

**DOI:** 10.1155/2016/2146204

**Published:** 2016-10-13

**Authors:** Jianping Lin, Shaoqing Chen, Shuzhen Li, Meili Lu, Yanan Li, Youxin Su

**Affiliations:** ^1^Subsidiary Rehabilitation Hospital, Fujian University of Traditional Chinese Medicine, Fuzhou, China; ^2^College of Rehabilitation Medicine, Fujian University of Traditional Chinese Medicine, Fuzhou, China; ^3^Orthopaedics and Traumatology College, Fujian University of Traditional Chinese Medicine, Fuzhou, China

## Abstract

*Background*. Chinese medicinal herbs may be useful for the treatment of hyperuricemia, but there has been no systematic assessment of their efficacy and safety.* Objectives*. To systematically assess the efficacy and safety of Chinese medicinal herbs for the treatment of hyperuricemia.* Methods*. Six electronic databases were searched from their inception to December 2015. Randomized controlled clinical trials (RCTs) were included. Cochrane criteria were applied to assess the risk of bias. Data analysis was performed using RevMan software version 5.2.* Results*. Eleven RCTs with 838 patients were included. There was no significant difference in serum uric acid between Chinese medicinal herbs and traditional Western medicine (SME: 0.19, 95% CI: −0.04 to 0.43; *p* = 0.10). In terms of overall efficacy, the Chinese medicinal herbs were significantly superior to Western medicine (RR: 1.11; 95% CI: 1.04 to 1.17; *p* = 0.0007). The Chinese medicinal herbs were better than Western medicine in reducing the adverse reactions (RR: 0.30; 95% CI: 0.15 to 0.62; *p* = 0.001). And all these funnel plots showed unlikelihood of publishing bias.* Conclusions*. The results indicate that Chinese medicinal herbs may have greater overall efficacy with fewer adverse drug reactions, although the evidence is weak owing to the low methodological quality and the small number of the included trials.

## 1. Introduction

Hyperuricemia (HUE) is a metabolic disease caused by a purine metabolic disturbance that leads to excessive production of uric acid, which is the pathogenic basis for gout. It has been reported that 18.8% of patients with HUE developed gout during a 5-year follow-up period [1]. Studies also show that, in addition to its association with gout, HUE is a strong risk factor for atherosclerosis [2], hypertension [3], and metabolic syndrome [4], which may include obesity, hyperlipidemia, and diabetes mellitus [5].

Rapid economic development in China has led to changes in the daily diet, specifically, an increased rate of high-purine diets, thereby increasing the prevalence of HUE. According to a meta-analysis conducted in 2011, the prevalence of HUE in China was 21.6% among men and 8.6% among women [6], while, in 2010, in the United States, it was 12.7% overall [7]. The many potential complications of HUE may lead to greatly increased healthcare costs. Therefore, it is extremely important to understand HUE not only in China but also around the world.

Because both excess production and decreased excretion of uric acid contribute to HUE, two types of hypouricemic drugs are commonly used for the treatment. Uricosuric drugs (i.e., probenecid and benzbromarone) that reduce the serum uric acid concentration (SUA) by increasing the renal excretion of uric acid and xanthine oxidase inhibitors (i.e., allopurinol) that decrease serum uric acid by inhibiting uric acid synthesis have been the mainstays of conventional HUE treatment [8]. Although these treatments have very good results as hypouricemic agents, they may be associated with gastrointestinal reactions, liver and kidney damage, and other adverse effects [9, 10], and, in rare cases, allopurinol has been associated with reactive hepatitis and fulminant hepatic failure [11]. Therefore, long-term treatment may not be advisable.

In traditional Chinese medicine (TCM), HUE belongs to the arthromyodynia disease category. Chinese medicinal herbs are administered orally as a liquid formulation prepared by chopping or crushing herbs into a thick powder, immersing the powder in water, boiling, and filtering [12], which has long been used for the treatment of HUE in China and has been associated with positive and unique clinical effects.

In the past decade, many studies have compared Chinese medicinal herbs with traditional Western medicine in the treatment of HUE. However, the treatment protocols and evaluation methodologies of these studies are different [24], which greatly limit their clinical applicability [25]. Therefore, a systematic review to evaluate the efficacy and safety of Chinese medicinal herbs in the treatment of HUE would be of great importance, and, to our knowledge, no such systematic review has been undertaken. We therefore designed this systematic review to investigate the efficacy and safety of Chinese medicinal herbs in the treatment of HUE.

## 2. Methods

### 2.1. Eligibility Criteria

Randomized controlled clinical trials (RCTs) in humans were included in this review. According to our study design, the included studies should focus on the effect of the Chinese medicinal herbs (single or compound) in comparison with Western medicine only. Outcome measures should include at least one essential outcome, such as SUA and overall efficacy.

### 2.2. Patients

In accordance with published diagnostic criteria, HUE [26] is diagnosed on the basis of SUA levels of up to 416 *μ*mol/L in men or 357 *μ*mol/L in women, with all other serum and uric influencing factors excluded. All patients included in the study had confirmed diagnoses of HUE. Pregnant women and patients with cancer or blood diseases were excluded.

### 2.3. Databases and Search Strategy

Original research articles were searched from 6 electronic databases from their inception to December 2015: PubMed, EMBASE, the Cochrane Library, the Chinese Scientific Journal Database (VIP), the China National Knowledge Information Database (CNKI), and Wanfang. The journal languages were restricted to Chinese and English.

We used the following search strategy: (“hyperuricemia”) AND (“traditional Chinese medicine” OR “Chinese medicine”, “traditional” OR “zhong yi xue” OR “Chinese Traditional Medicine” OR “Traditional Medicine”, “Chinese” OR “Drugs”, “Chinese Herbal” OR “herbal medicine” OR “Chinese medicine practice”) AND (“control” OR “comparison” OR “controlled trial”) in the English databases and (“hyperuricemia” AND “Chinese Herbal” AND “comparison”) in the Chinese databases.

### 2.4. Endpoint Indicators

Enumeration data of the efficacy were graded into 4 categories according to the standards of the Guiding Principles for the Clinical Research of New TCM [27]: cure, SUA decreased to normal level; markedly effective, the rate of decrease of SUA was equal to or more than 30%; effective, the rate of decrease of SUA was 5% to 30%; and ineffective, the rate of decrease of SUA was less than 5%. The rate of decrease of SUA is equal to (SUA before intervention minus SUA after intervention) divided by SUA before intervention · 100%. Enumeration data for effective treatment were counted in each group based on the categories of cured, markedly effective, and effective. Measurement data included SUA as a primary indicator.

### 2.5. Study Identification

Three investigators participated in the data extraction from all publications included in the study. Data including the first author, publication year, total number of cases included in the experimental and control groups, intervention methods, and endpoint evaluation indicators were recorded. Two reviewers (LJP and CSQ) independently screened the title and abstract of the searched studies. Full text of the studies that potentially met the eligibility criteria were obtained, and the potentially relevant references were retrieved according to predefined eligibility criteria. One investigator (LJP) performed the initial data extraction, and a second investigator (CSQ) subsequently reexamined each article and verified the results. Differences were resolved by discussion with the third investigator (SYX) in order to reach consensus. And the original author was contacted if the results could not come to an agreement.

### 2.6. Quality Assessment

The quality of the included studies was assessed independently by two reviewers (LZP and LSZ) using the Cochrane Collaboration tool for assessing the risk of bias [28]. When there were inconsistencies, a third reviewer (CSQ) participated in the assessment and consensus was reached by discussion. The Cochrane-recommended domains that were considered were selection bias, which is a measure of adequate sequence generation and allocation concealment; performance bias, which evaluates blinding of participants and personnel; detection bias, which checks for blinding of outcome assessment; attrition bias, which checks for incomplete outcome data; reporting bias, which indicates selective outcome reporting; and other bias, which covers other sources of bias including baseline imbalance and fraudulent outcomes. Each of these indicators was scored as low risk of bias, high risk of bias, or unclear [29].

### 2.7. Data Analysis

We calculated the *d* index and the standard error (SE_*d*_) values of SUA for each RCT before using RevMan. The *d* index and SE_*d*_ values of SUA were continuous, and the standardized mean difference (SMD) and 95% confidence interval (CI) were calculated. Enumeration data (overall efficacy and adverse reactions) were evaluated by relative risk (RR) and 95% CI. Statistical heterogeneity was assessed using a Chi-square test or by calculating Higgins *I* ^2^ values [29]. The proper effect models were chosen in accordance with the results: when *I* ^2^ is less than 50%, fixed effects models were chosen, and when *I* ^2^ is more than 50%, a random effects model was applied [30]. Sensitivity analysis was used to explore the source of heterogeneity. The Cochrane Collaboration Review Manager Software (RevMan version 5.2.0) was used for all statistical analyses, and all *p* values were two sided [29].

## 3. Results

### 3.1. Description of Studies

**Figure 1:**
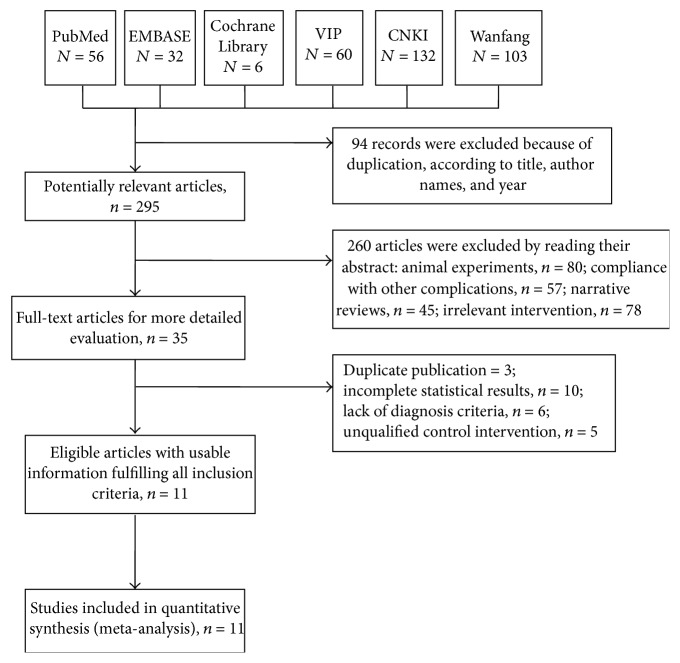
Flowchart of the included articles. Abbreviations: VIP: Chinese Scientific Journal Database; CNKI: China National Knowledge Information Database.

The study selection flowchart is presented in Figure 1. A total of 389 records were identified through the database searches. After removing duplicates, 295 studies remained for eligibility screening; 284 of them were ultimately excluded, including 80 that were based on animal experiments, 57 that complied with other complications, 45 that were narrative reviews, 78 that considered irrelevant interventions, 3 that were duplicate publications, 10 that had incomplete statistical results, 6 that did not meet the diagnostic criteria, and 5 that had unqualified control interventions. Thus, 11 articles met the inclusion criteria, and 11 were included in the meta-analysis [13–23].

### 3.2. Serum Uric Acid Concentration

**Figure 2:**
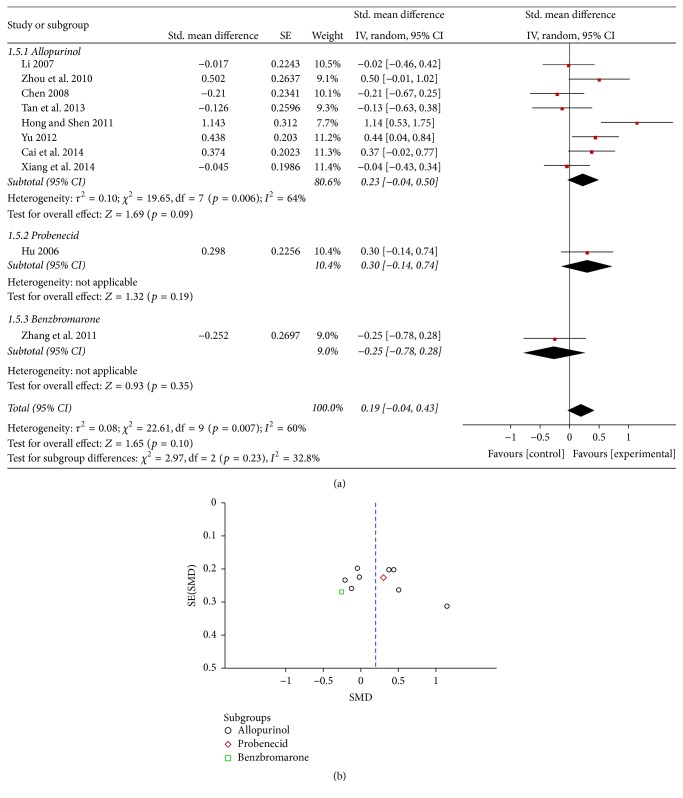
(a) Effects of Chinese medicinal herbs on serum uric acid in hyperuricemic patients. (b) A funnel plot: effects of Chinese medicinal herbs on serum uric acid in hyperuricemic patients.

Ten RCTs provided SUA data in *μ*mol/L. These studies included 766 patients (393 patients in the experimental groups and 373 in the control groups) [14–23]. The subgroup meta-analysis showed that 2 RCTs were statistically significant differences between the TCM Chinese medicinal herbs and allopurinol (SMD 1.14, 95% CI: 0.53 to 1.75 and SMD 0.44, 95% CI: 0.04 to 0.84) because *I* ^2^ = 60%, a random effects model was used for the analysis, and the combined SMD was 0.19 with a 95% CI of −0.04 to 0.43 (*p* = 0.10). Therefore, there was no significant difference between the Chinese medicinal herbs and the Western pharmaceuticals in terms of SUA reduction (Figure 2(a)). The funnel plot showed symmetrical distribution in 10 experiments, indicating unlikelihood of publishing bias (Figure 2(b)).

### 3.3. Overall Efficacy of the Chinese Medicinal Herbs

**Figure 3 fig3:**
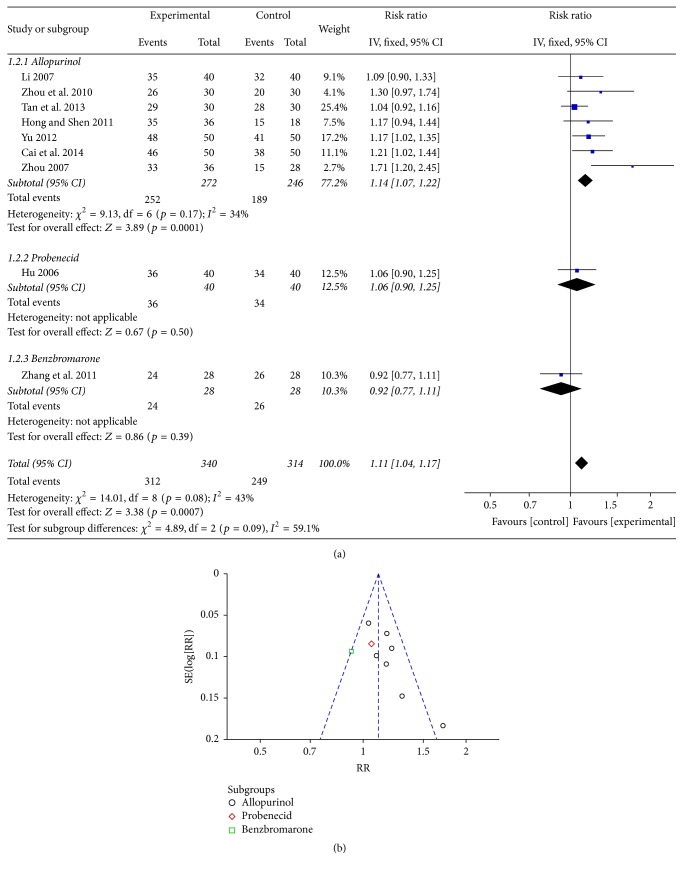
(a) An analysis of the overall efficacy of Chinese medicinal herbs and Western medicine in hyperuricemic patients. (b) A funnel plot: an analysis of the overall efficacy of Chinese medicinal herbs and Western medicine in hyperuricemic patients.

Nine RCTs were analyzed, including 654 patients (340 in the experimental groups and 314 in the control groups) (Table 2) [13, 15–17, 19–23]. On subgroup meta-analysis, 3 RCTs showed statistically significant differences between the TCM Chinese medicinal herbs and allopurinol (SMD 1.17, 95% CI: 1.02 to 1.35; SMD 1.21, 95% CI: 1.02 to 1.44; and SMD 1.71, 95% CI: 1.20 to 2.45). For all studies, the meta-analysis indicated that *I* ^2^ = 43%, and a fixed effects model was adopted for the analysis. The combined RR was 1.11, and the 95% CI was 1.04 to 1.17 (*p* = 0.0007), indicating a significant difference between the overall efficacy of Chinese medicinal herbs and Western pharmaceuticals for the treatment of HUE (Figure 3(a)). The funnel plot showed 9 symmetric distributions of 9 experiments, indicating unlikelihood of publishing bias (Figure 3(b)).

### 3.4. Adverse Reactions

**Figure 4:**
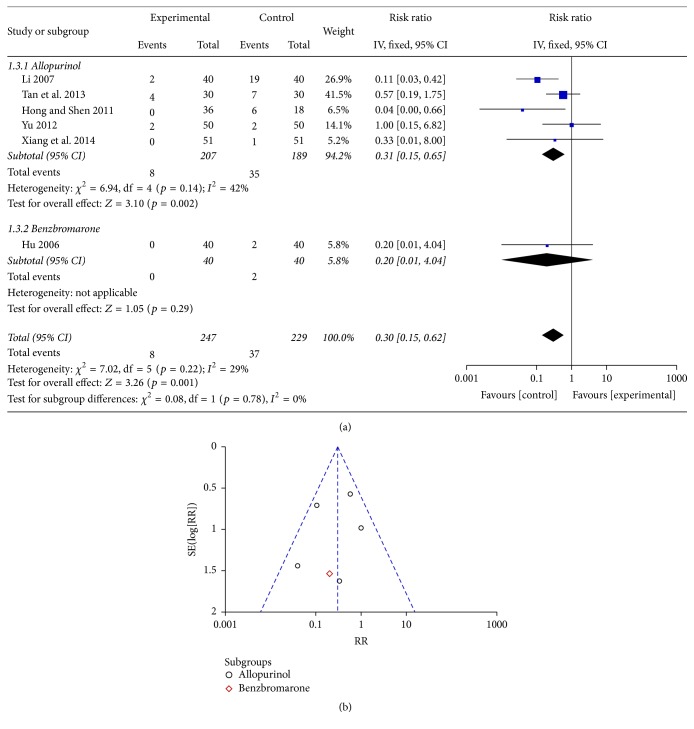
(a) An analysis of the adverse reactions of Chinese medicinal herbs and Western medicine in hyperuricemic patients. (b) A funnel plot: an analysis of the adverse reactions of Chinese medicinal herbs and Western medicine in hyperuricemic patients.

Six RCTs including 476 patients (247 in the experimental groups and 229 in the control groups) provided safety evaluation data [15–18, 21, 22]. Subgroup meta-analysis showed statistical significance in the allopurinol subgroups (RR: 0.31 and 95% CI: 0.15 to 0.65; *p* = 0.002). The meta-analysis showed that *I* ^2^ for all 6 studies was 29%, and the analysis was performed using a fixed effects model. The combined RR value was 0.30 and the 95% CI was 0.15 to 0.62 (*p* = 0.001), indicating that, among the studies included, there were fewer adverse reactions in patients using Chinese medicinal herbs (Figure 4(a)). The funnel plot showed 6 symmetric distributions of 6 experiments, indicating unlikelihood of publishing bias (Figure 4(b)).

### 3.5. Methodological Quality

**Figure 5:**
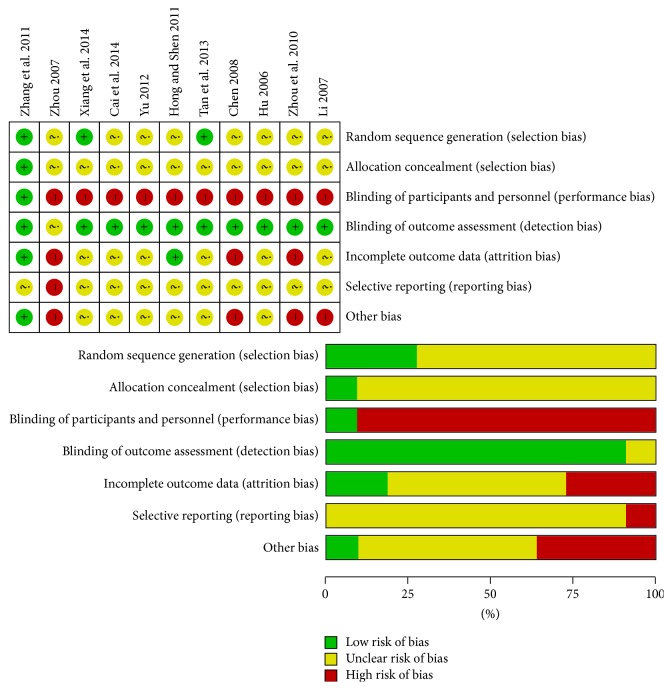
Risk of bias summary and graph.

Among 11 included RCTs, 8 reported no significant differences at baseline between patients in the Chinese medicinal herbs groups and those in the Western medicine groups. Only 3 trials described a randomization technique (random sequence generation using a random number table), while 8 did not describe the randomization technique and 10 failed to describe concealed random allocation. Although only 1 trial had a double-blind design, the outcome measurements of other 9 trials relied on objective indicators that were not expected to be influenced by lack of blinding (Figure 5).

### 3.6. Sensitivity Analysis

The results of sensitivity analysis were relatively stable through performance from a fixed effects model to a random effects model (Figures 2 [Fig fig3]–4).

## 4. Discussion

This systematic review included 11 RCTs with 838 total patients comparing Chinese medicinal herbs with Western medicine for the treatment of HUE. The outcome measures included change in SUA values, overall efficacy, and adverse reactions. Our analysis showed that Chinese medicinal herbs and traditional Western medicine had a similar effect for reducing SUA values, but, in terms of overall treatment efficacy and adverse reactions, there were significant advantages for the Chinese medicinal herbs.

The above results showed that it could not just observe SUA values, but overall treatment efficacy should be considered in order to systematically assess the efficacy and safety of Chinese medicinal herbs for the treatment of hyperuricemia. The enumeration data of the efficacy were graded into 4 categories according to the standards of the Guiding Principles for the Clinical Research of New TCM, which were advantageous to the standardization of TCM research. However, it needs more argumentation and research to get internationally recognized [31]. Therefore, no definitive conclusion can be drawn that Chinese medicinal herbs have greater overall efficacy than Western medicine. And additional studies will be needed in the future.

The results indicate that Chinese medicinal herbs have a positive therapeutic effect with fewer adverse drug reactions. We believe that the main reason for the efficacy of Chinese medicinal herbs in the treatment of HUE relates to the active ingredients of these extracts [32]. The bulbs of* pseudobulb cremastra seu pleiones*, which is an ingredient of decoctions used by Li [17], Zhang et al. [23], and others, contain colchicine [33]. In addition, the Chinese medicinal herbs used by Zhou [13], Yu [15], Tan et al. [16], and Li [17] contain* glabrous greenbrier rhizome* (Table 1), which has astilbin as an active ingredient. Astilbin has been associated with increased renal blood flow and has shown anti-inflammatory and analgesic actions.* Plantago* contains the active ingredient aucubin [34], which Chen [14], Tan et al. [16], Li [17], and Zhang et al. [23] believe has a significant effect toward facilitating urine production and promoting the excretion of urea and chloride [35]. Moreover, other herbs have been used, such as seven-lobed yam rhizome [36], jobstears seed [37], and* radix achyranthis bidentatae* [38], all of which enhancing additional functions including promotion of uric acid excretion, reduction of platelet accumulation resulting in improved microcirculation, and anticoagulant function. The actions of these ingredients contribute to reductions in SUA and amelioration of potential side effects during treatment.

Traditional Western medicines such as allopurinol, probenecid, and benzbromarone have been cornerstones in the treatment of HUE and gout for decades [39]. Allopurinol is an analogue of hypoxanthine, which inhibits xanthine oxidase. In most patients, it is well tolerated. However, about 2% of patients will develop a skin rash, and allopurinol has been associated with rare life-threatening hypersensitivity syndromes at a rate of approximately 4/1000 cases [40–42]. Probenecid and benzbromarone, the uricosuric drugs, may lead to renal tubular aggregation of urate crystals and induce renal damage. Finally, in 2003, benzbromarone was withdrawn from the market because of serious hepatotoxicity. Although it is still marketed in several countries by other drug companies, withdrawal by the French manufacturer Sanofi-Synthélabo has greatly limited its availability around the world [43]. As per physicians practicing TCM, HUE is a condition that is caused by a congenital deficiency and a circulatory blockade. Blood stasis in combination with phlegm and exogenous pathogenic factors blocks the meridians and collaterals or even goes deep to the bone, transforms to heat, and finally causes renal damage [44]. Thus, Chinese herbal prescriptions usually contain several herbal components for dissipating blood stasis and reinforcing the kidneys, and these herbal components work synergistically [45]. For example, Tan et al. [16] and others add* radix achyranthis bidentatae* and* Salvia miltiorrhiza* Bge. to the prescription, which serves to inhibit platelet aggregation, expand small arteries, improve microcirculation, inhibit atherosclerotic lesions, ameliorate renal fibrosis, and protect the kidneys [37, 46]. These benefits may be among the reasons that the active ingredients in Chinese medicinal herbs are able to effectively reduce the potentially adverse effects.

Most evaluations of Chinese medicinal herbs that reduce SUA have focused on the active ingredients of individual herbs. However, Chinese medicinal herbs are usually compounded in a liquid decoction. Because Chinese herbal formulations may contain several herbal components, the active ingredients of each should be considered [47]. Furthermore, many of the available reports concerned animal experiments, and large-scale clinical trials in humans were lacking. Therefore, additional studies in humans should be a goal in the future.

The present systematic review has limitations, mainly because of the low methodological quality and small number of the included trials. Indeed, most of the included RCTs were of poor quality. Of 11 trials, only 3 described a randomization method, only 1 had a double-blind design, and none described the calculation of the sample size. Moreover, no multicenter large-scale RCT met our inclusion criteria. The low quality of the research included in this review may lead to some overestimation of the overall efficacy of Chinese medicinal herbs in comparison to traditional Western medicines (Figure 5). Furthermore, only 6 of the 11 RCTs described adverse reactions. Owing to insufficient descriptions, enumeration data for adverse reactions included only renal and hepatic dysfunction, skin reaction, gastrointestinal reaction, and acute arthritis. Therefore, a more rational approach for the evaluation of adverse drug reactions from Chinese medicinal herbs with the development of large-scale and well-designed RCTs will be needed in the future.

## 5. Conclusion

The results of this review indicate that Chinese medicinal herbs have a positive therapeutic effect in the treatment of HUE and can safely reduce SUA while ameliorating adverse effects. Because of the low methodological quality and the small number of the included trials, no definitive conclusion can be drawn at this point, and these results should be interpreted cautiously. Additional large-scale, well-designed trials are needed.

## Figures and Tables

**Table 1:** Characteristics of the included studies.

Author, year	Sample size (EG^▲^)	Sample size (CG^▲^)	Age (EG)	Age (CG)	Disease severity (EG)	Disease severity (CG)	Intervention method (/d) (EG)	Intervention methods (/d) (CG)	Duration treatment (d) (EG)	Duration treatment (d) (CG)
Zhou, 2007 [13]	36	36	55.2 ± 6.1	53.8 ± 6.3	SUA^■^ (umol/L): 518.2 ± 56.4	SUA (umol/L): 518.6 ± 46.8	Swordlike atractylodes (15 g), largehead atractylodes rhizome (15 g), Indian buead (15 g), plantain seed (10 g), etc.	Allopurinol (0.1 g *∗* 3)	21	21
Chen, 2008 [14]	38	36	22~65	22~65	N/A^★^	N/A	Glabrous greenbrier rhizome (45 g), seven-lobed yam rhizome (30 g), *Carica papaya* (15 g), plantain seed (15 g), etc.	Allopurinol 0.2 g	28	28
Yu, 2012 [15]	50	50	48.12 ± 10.2	47.12 ± 11.5	SUA (umol/L) (585.83 ± 93.93)	SUA (umol/L) (584.94 ± 90.29)	Glabrous greenbrier rhizome (50 g), herba lysimachiae (50 g), cortex phellodendri chinensis (15 g), swordlike atractylodes (15 g), etc.	Allopurinol (0.1 g *∗* 2)	30	30
Tan et al., 2013 [16]	30	30	44.17 ± 8.27	45.23 ± 9.35	The course of disease: (8.54 ± 4.07) years	The course of disease: (8.10 ± 3.46) years	Glabrous greenbrier rhizome (15 g), seven-lobed yam rhizome (20 g), jobstears seed (10 g), herba lysimachiae (15 g), etc.	Allopurinol (0.1 g *∗* 3)	56	56
Li, 2007 [17]	40	40	55~81	52~78	N/A	N/A	Jobstears seed (30 g), glabrous greenbrier rhizome (30 g), plantain deed (20 g), seven-lobed yam rhizome (20 g), etc.	Allopurinol (0.1 g *∗* 3)	60	60
Xiang et al., 2014 [18]	51	51	45.62 ± 5.05	44.72 ± 5.58	The course of disease: (1.96 ± 0.45) years	The course of disease: (1.13 ± 0.46) years	Radix clematidis (60 g), rhizoma polygonati odorati (20 g), radix achyranthis bidentatae (15 g)	Allopurinol (0.05 g *∗* 3)	90	90
Cai et al., 2014 [19]	50	50	48 ± 10	47 ± 12	SUA (umol/L) (517.2 ± 86.33)	SUA (umol/L) (514.5 ± 90.29)	Cortex phellodendri chinensis (15 g), swordlike atractylodes (15 g), plantain seed (10 g), oriental waterplantain rhizome (10 g), etc.	Allopurinol (0.1 g *∗* 3)	28	28
Zhou et al., 2010 [20]	30	30	35–74	32–75	N/A	N/A	Glabrous greenbrier rhizome (40 g), cortex phellodendri chinensis (15 g), swordlike atractylodes (15 g), jobstears seed (20 g), etc.	Allopurinol (0.1 g *∗* 2)	28	28
Hong and Shen, 2011 [21]	36	18	51.8 ± 6.6	49.7 ± 5.1	SUA (umol/L) (546.0 ± 78.1)	SUA (umol/L) (533.0 ± 62.31)	Glabrous greenbrier rhizome (15 g), cortex phellodendri chinensis (10 g), swordlike atractylodes (10 g), jobstears seed (30 g), etc.	Allopurinol (0.1 g *∗* 3)	60	60
Hu, 2006 [22]	40	40	39.38 ± 10.89	40.88 ± 11.13	The course of disease: (2.64 ± 1.96) years, hypertension: 11, diabetes: 4, and hyperlipidaemia: 17	The course of disease: (2.69 ± 1.86) years, hypertension: 14, diabetes: 1, and hyperlipidaemia: 15	Glabrous greenbrier rhizome (30 g), jobstears seed (15 g), oriental waterplantain rhizome (15 g), herba lysimachiae (20 g), etc.	Probenecid (0.25*∗*2)	28	28
Zhang et al., 2011 [23]	28	28	56.07 ± 17.62	53.18 ± 16.40	Hypertension: 9, diabetes: 7, hyperlipidaemia: 6, and coronary heart disease: 5	Hypertension: 11, diabetes: 8, hyperlipidaemia: 7, and coronary heart disease: 5	Glabrous greenbrier rhizome (35 g), seven-lobed yam rhizome (18 g), pseudobulb cremastra seu pleiones (15 g), radix achyranthis bidentatae (10 g), etc.	Benzbromarone (50 mg *∗* 1)	20	20

^▲^EG: experimental group; ^▲^CG: control group.

^★^N/A: not applicable.

^■^SUA: serum uric acid concentration.

**Table 2:** Outcomes.

Author, year	The overall efficacy (EG^▲^) C/M/E/I^☆^	The overall efficacy (CG^▲^) C/M/E/I^☆^	The overall efficacy (RR, 95% CI); *p* value	SUA^●^ (umol/L) (EG: B/A^■^)	SUA (umol/L) (CG:B/A)	SUA (SMD, 95% CI); *p* value	Adverse reactions (EG)	Adverse reactions (CG)	Adverse reactions (RR, 95% CI)
Zhou, 2007 [13]	16/0/17/3^☆^	5/0/10/13	1.71 (1.20, 2.45); *p* < 0.05	N/A^★^	N/A	N/A	N/A	N/A	N/A
Chen, 2008 [14]	N/A	N/A	N/A	488.91 ± 70.51/316.12 ± 86.25	489.61 ± 69.89/301.93 ± 93.77	0.16 (−0.30, 0.61); *p* > 0.05	N/A	N/A	N/A
Yu, 2012 [15]	0/12/36/2	0/8/33/9	1.17 (1.02, 1.35); *p* < 0.01	585.83 ± 93.93/319.13 ± 87.63	584.94 ± 90.29/358.86 ± 88.53	−0.45 (−0.84, −0.05); *p* < 0.05	Diarrhea: 2	Liver damaged: 2	1.00 (0.15, 6.82)
Tan et al., 2013 [16]	0/15/14/1	0/17/11/2	1.04 (0.92, 1.16); *p* = 0.266	546.69 ± 51.45/354.72 ± 27.29	550.33 ± 45.99/352.14 ± 25.67	0.10 (−0.41, 0.60); *p* = 0.000	Liver damaged: 1, kidney damaged: 1, leucopenia: 0, nausea: 2, and acute arthritis: 0	Liver damaged: 2, kidney damaged: 1, leucopenia: 1, nausea: 1, and acute arthritis: 2	0.57 (0.19, 1.75)
Li, 2007 [17]	0/21/14/5	0/19/13/8	1.09 (0.90, 1.33); *p*: N/A	524.43 ± 43.14/410.17 ± 60.25	528.50 ± 45.72/413.46 ± 55.84	−0.06 (−0.49, 0.38); *p* < 0.05	Diarrhea: 2	Liver damaged: 10, leucopenia: 8, and itchy skin: 1	0.11 (0.03, 0.42)
Xiang et al., 2014 [18]	N/A	N/A	N/A	451.33 ± 98.55/370.45 ± 91.45	442.64 ± 97.46/357.35 ± 98.57	0.14 (−0.25, 0.53); *p* < 0.05	None	Itchy skin: 1	0.33 (0.15, 0.65)
Cai et al., 2014 [19]	11/0/35/4	6/0/32/12	1.21 (1.02, 1.44); *p* < 0.05	517.2 ± 86.33/328.8 ± 87.56	514.5 ± 90.29/359.4 ± 88.63	−0.34 (−0.74, 0.05); *p* < 0.01	N/A	N/A	N/A
Zhou et al., 2010 [20]	0/14/12/4	0/8/12/10	1.30 (0.97, 1.74); *p* < 0.05	521.84 ± 75.32/290.45 ± 74.76	514.43 ± 83.35/323.44 ± 71.98	−0.44 (−0.96, 0.07); *p* < 0.05	N/A	N/A	N/A
Hong and Shen, 2011 [21]	29/0/6/1	7/0/8/3	1.17 (0.94, 1.44); *p* < 0.05	546.0 ± 78.1/353.0 ± 122.6	533.0 ± 62.3/425.0 ± 169.1	−0.51 (−1.08, 0.07); *p* < 0.05	None	Liver damaged: 3, abnormal changes of blood cells: 2, and acute episode of gout: 1	0.04 (0.00, 0.66)
Hu, 2006 [22]	0/20/16/4	0/22/12/6	1.06 (0.90, 1.25); *p* > 0.05	575.38 ± 52.62/389.30 ± 13.07	568.48 ± 53.10/398.30 ± 610.08	−0.20 (−0.64, 0.24); *p* > 0.05	None	Nausea: 1 and itchy skin: 1	0.2 (0.01, 4.04)
Zhang et al., 2011 [23]	0/14/10/4	0/18/8/2	0.92 (0.77, 1.11); *p* > 0.0167	508.00 ± 89.46/386.57 ± 69.60	493.71 ± 65.45/352.21 ± 66.79	0.5 (−0.04, 1.03); *p* > 0.05	None	N/A	N/A

^☆^C/M/E/I: cure/markedly effective/effective/ineffective.

^▲^EG: experimental group; ^▲^CG: control group.

^■^B/A: before intervention/after intervention.

^★^N/A: not applicable.

^●^SUA: serum uric acid concentration.

## References

[1] Lin K.-C., Lin H.-Y., Chou P. (2000). The interaction between uric acid level and other risk factors on the development of gout among asymptomatic hyperuricemic men in a prospective study. *The Journal of Rheumatology*.

[2] Kawamoto R., Tomita H., Oka Y., Kodama A., Kamitani A. (2006). Metabolic syndrome amplifies the LDL-cholesterol associated increases in carotid atherosclerosis. *Internal Medicine*.

[3] Johnson R. J., Kang D.-H., Feig D. (2003). Is there a pathogenetic role for uric acid in hypertension and cardiovascular and renal disease?. *Hypertension*.

[4] Wei C.-Y., Sun C.-C., Wei J. C.-C. (2015). Association between hyperuricemia and metabolic syndrome: an epidemiological study of a labor force population in Taiwan. *BioMed Research International*.

[5] Chien K.-L., Chen M.-F., Hsu H.-C. (2008). Plasma uric acid and the risk of type 2 diabetes in a Chinese community. *Clinical Chemistry*.

[6] Liu B., Wang T., Zhao H. N. (2011). The prevalence of hyperuricemia in China: a meta-analysis. *BMC Public Health*.

[7] Krishnan E. (2014). Interaction of inflammation, hyperuricemia, and the prevalence of hypertension among adults free of metabolic syndrome: NHANES 2009-2010. *Journal of the American Heart Association*.

[8] Wu T.-H., Chen L.-C., Yang L.-L. (2007). Hypouricemic effect and regulatory effects on autonomic function of Shao-Yao Gan-Cao Tang, a Chinese herbal prescription, in asymptomatic hyperuricemic vegetarians. *Rheumatology International*.

[9] Arroyo M. P., Sanders S., Yee H., Schwartz D., Kamino H., Strober B. E. (2004). Toxic epidermal necrolysis-like reaction secondary to colchicine overdose. *British Journal of Dermatology*.

[10] Ozdogu H., Boga C., Yilmaz Z., Sahin F. I., Bal N. (2007). Long-term colchicine therapy in a patient with Behçet's disease and acute promyelocytic leukemia. *Rheumatology International*.

[11] Yoon J. Y., Min S. Y., Park J. Y. (2008). A case of allopurinol-induced granulomatous hepatitis with ductopenia and cholestasis. *Korean Journal of Hepatology*.

[12] Ying Y. (2009). To explore traditional Chinese medicine decoction and Fried condition. *China Medical Herald*.

[13] Zhou W. (2007). Treatment of 36 cases of hyperuricacidemia by Pingwei Powder. *Journal of Zhejiang University of Chinese Medicine*.

[14] Chen L. (2008). Treatment of 38 cases of hyperuricacidemia by resolving turbidity and eliminating dampness. *Yunnan Journal of Traditional Chinese Medicine and Materia Medica*.

[15] Yu Q. (2012). Treatment of 100 cases of hyperuricacidemia by clearing heat, detoxicating eliminating dampness and removing the obstruction in meridians. *Chinese Journal of Gerontology*.

[16] Tan N., Huang S., Luo X., Zhu H., Liao K. (2013). Effects of chusi huayu prescription on endothelin-1 (ET-1)and plasminogen activator inhibitor-1(PAI-1) of hperuricemia patients. *Liaoning Journal of Traditional Chinese Medicine*.

[17] Li H. (2007). The clinical observation of decreasing serum uric acid decoction for treatment of primary hyperuricemic elderly men. *Chinese Journal of Gerontology*.

[18] Xiang T., Sun B., Zhang S. (2014). Treatment of 51 cases of and heat syndrome of primary hyperuricacidemia by decreasing serum uric acid decoction. *Jiangxi journal of traditionla chinese*.

[19] Cai S., Xu Z., Chen W. (2014). The clinical observation of clearing heat, eliminating dampness and removing the obstruction in meridians for the treatment of hyperuricacidemia. *Clinical Journal of Traditional Chinese Medicine*.

[20] Zhou J., Liu C., Wei Y., Xue T. (2010). Treatment of 60 cases of primary by Gout fourteen flavour decoction. *Shaanxi Journal of Traditional Chinese*.

[21] Hong Q., Shen P. (2011). Treatment of 36 cases of primary hyperuricacidemia by warming meridian, dispelling cold and removing dampness. *Shanghai Journal of Traditional Chinese Medicine*.

[22] Hu M. (2006). The clinical efficacy of Strengthening spleen, Resolving phlegm, dredging collaterals and excreting dampness. *Yunnan Journal of Traditional Chinese Medicine and Materia Medica*.

[23] Zhang X., Sun W., Xu W., Wang T. (2011). Assessment on the clinical efficacy and safety of Xiezhuo Chubi recipe in treating hyperuricemla. *Chinese Journal of Integrated Traditional and Western Medicine*.

[24] Li T., Liu X., Zhang M., Ma J. (2007). Assessment of systematic reviews and meta-analyses on traditional Chinese medicine published in Chinese journals. *Chinese Journal of Evidence-Based Medicine*.

[25] Huedo-Medina T. B., Sánchez-Meca J., Marín-Martínez F., Botella J. (2006). Assessing heterogeneity in meta-analysis: *Q* statistic or *I*
^2^ index?. *Psychological Methods*.

[26] Liao E. Y., Mo C. H. (2002). *Endocrinology*.

[27] The Ministry of Health of the People's Republic of China Chinese herbal medicine new medicine clinical research guiding principle.

[28] Savović J. J., Weeks L., Sterne J. A. C. (2014). Evaluation of the Cochrane Collaboration's tool for assessing the risk of bias in randomized trials: focus groups, online survey, proposed recommendations and their implementation. *Systematic Reviews*.

[29] Zheng G., Li S., Huang M., Liu F., Tao J., Chen L. (2015). The effect of Tai Chi training on cardiorespiratory fitness in healthy adults: a systematic review and meta-analysis. *PLoS ONE*.

[30] Zhou L., Liu L., Liu X. (2014). Systematic review and meta-analysis of the clinical efficacy and adverse effects of Chinese herbal decoction for the treatment of gout. *PLoS ONE*.

[31] Xu J., Xu Z., Wang Y., Yan H., Xu W. (2014). Traditional Chinese medicine syndrome standardization research progress based on the combination of disease and syndrome standardization. *China Journal of Traditional Chinese Medicine and Pharmacy*.

[32] Wang H., Zhan J., Wang X., Zou L. (2015). Research progress in treatment of hyperuricemia with active ingredients of traditional Chinese medicine. *Chinese Journal of Pharmacology and Toxicology*.

[33] Tian S., Chen S., Chen Z. (2002). To optimize the extracting colchicine of Pseudbubus Cremastra Seu pleiones. *Chinese Traditional and Herbal Drugs*.

[34] Li Q. (2008). Research of Glabrous Greenbrier Rhizome. *Journals of China Pharmaceutical*.

[35] Zeng J., Bi Y., Wei J. (2013). The research of Plantago asiatica L. herbs extracts reduce the level of uric acid in hyperuricemia mice and it’s mechanism. *Lishizhen Medicine and Materia Medica Research*.

[36] Chen G., Liu H., Han R., Xu S. (2007). Studies on the rationality of TSD combined with TSA on reducing serunl uric acid and anti-inflammatory in mice. *Chinese Pharmacological Bulletin*.

[37] Li H., Wen C., Xie Z., Han C., Wang M. (2013). Literature research of traditional chinese medical prescriptions of gout in intermission and chronic phase. *Chinese Archives of Traditional Chinese Medicine*.

[38] Jun Z. (2009). The research of chemical compositions and pharma-cological effects of Radix Achyranthis Bidentatae. *Tianjin Pharmacy*.

[39] Gutiérrez-Macías A., Lizarralde-Palacios E., Martínez-Odriozola P., Miguel-De La Villa F. (2005). Fatal allopurinol hypersensitivity syndrome after treatment of asymptomatic hyperuricaemia. *British Medical Journal*.

[40] Singer J. Z., Wallace S. L. (1986). The allopurinol hypersensitivity syndrome: unnecessary morbidity and mortality. *Arthritis & Rheumatism*.

[41] Arellano F., Sacristán J. A. (1993). Allopurinol hypersensitivity syndrome: a review. *Annals of Pharmacotherapy*.

[42] Kumar A., Edward N., White M. I., Johnston P. W., Catto G. R. D. (1996). Allopurinol, erythema multiforme, and renal insufficiency. *British Medical Journal*.

[43] Lee M.-H. H., Graham G. G., Williams K. M., Day R. O. (2008). A benefit-risk assessment of benzbromarone in the treatment of gout. Was its withdrawal from the market in the best interest of patients?. *Drug Safety*.

[44] Chen Q., Ma L., Akebaier W. (2009). Clinical study on treatment of hyperuricaemia by retention enema of Chinese herbal medicine combined with allopurinol. *Chinese Journal of Integrative Medicine*.

[45] Zhao J., Wang S., Fu Q., Wu W. (2014). Traditional Chinese medicine treatment of high uric acid nephropathy and it's advances. *Drugs and Clinic*.

[46] Liu Y., Meng Z., Zhang C., Yang Z. (2013). Study on the anti-hyperuricemia effect of Salvia miltiorrhiza Bge. *Strait Pharmaceutical Journal*.

[47] Zhang C., Li J.-L., Tian J.-X., Tong X.-L. (2013). Research progress of treating hyperuricemia by traditional Chinese medicine. *Chinese Journal of New Drugs*.

